# Retreatment of Patients Nonresponsive to Pegylated Interferon and Ribavirin with Daily High-Dose Consensus Interferon

**DOI:** 10.1155/2010/537827

**Published:** 2010-10-10

**Authors:** Douglas F. Meyer, Hillel Tobias, Albert D. Min, Arathi Rajendra, Ivanka Zic, Edward Brettholz, David J. Clain, Franklin Klion, David Bernstein, Henry C. Bodenheimer

**Affiliations:** ^1^Division of Digestive Diseases, Beth Israel Medical Center and Albert Einstein, College of Medicine, New York, NY 10003, USA; ^2^Division of Gastroenterology, New York University Medical Center, New York, NY 10016, USA; ^3^Division of Liver Diseases, Mount Sinai School of Medicine, New York, NY 10029, USA; ^4^Division of Gastroenterology, Hepatology and Nutrition, North Shore University Hospital, Manhasset, NY 11030, USA

## Abstract

*Background*. Current treatment of chronic hepatitis C with pegylated interferon and ribavirin has the ability to eliminate viral infection in about half of the patients treated. Therapeutic options, for those with remaining chronic hepatitis, will remain limited until novel antivirals become available in the future. Consensus interferon is currently available and has demonstrated clinical efficacy with superior *invitro* antiviral activity, but the maximum tolerated dose is not defined. *Methods*. We assessed the efficacy of daily high-dose (24 ug) consensus interferon with weight-based (1000–1200 mg daily) ribavirin in HCV genotype 1-infected non-responder patients. *Results*. Six adverse events were documented in five patients, and the trial was terminated with no subject achieving viral clearance. *Conclusions*. The occurrence of serious adverse events effectively defined the upper limit of acceptable dose, while also revealing that this dose did not offer enhanced sustained viral clearance.

## 1. Introduction

The most commonly employed treatment of patients with hepatitis C virus (HCV) infection who are treatment-naïve consists of pegylated interferon-alfa and ribavirin with sustained viral response (SVR) rates in genotype 1 patients of up to 52% [1, 2]. Since the FDA approval of pegylated interferon and ribavirin, there are an ever-increasing number of hepatitis C patients who have failed to respond to this combination therapy. There is no standard of care for the treatment of patients who have failed to respond to pegylated interferon and ribavirin [3]. Consensus interferon (CIFN), in combination with ribavirin, has recently been approved for this use. Patients with advanced fibrosis may not have time to await development of future antiviral agents and may need to utilize currently available therapies.

CIFN has more potent antiviral *invitro* activity compared to other interferons [4, 5]. Currently, CIFN is approved for initial or retreatment of persons infected with HCV. Recent clinical trials report reasonable SVR rates (23% to 37%) in the treatment of nonresponders to standard or pegylated interferon and ribavirin using different regimens of daily CIFN and weight-based ribavirin dosage [6–8]. Cornberg et al. found that an eight-week induction phase with CIFN doses of 18 *μ*g followed by 9 *μ*g for 40 weeks did not result in a higher SVR compared to 9 *μ*g for 48 weeks, presumably due to dose modifications in subjects receiving the induction dose of CIFN. When the subgroup of patients receiving the higher dose of CIFN, without early dose modification, was evaluated, 40% achieved SVR [8]. There was one multicenter randomized control trial, the DIRECT trial, evaluating the efficacy of daily CIFN at doses of 9 *μ*g or 15 *μ*g with weight-based ribavirin administration in the treatment of patients unresponsive to prior pegylated interferon and ribavirin treatment. Viral clearance was achieved in a dose-dependent manner with greatest loss of HCV in the 15 *μ*g group [9]. Although this trial suggested a dose-dependent rate of viral clearance, there has been no dose-ranging information to set the maximum tolerated dose of daily CIFN in this difficult-to-treat group of hepatitis C patients. Given the urgent need of treatment for patients with advanced fibrosis and limited access to antiviral agents under development, evaluation of available agents in novel dosing regimens is needed. 

The aim of our clinical trial was to evaluate the efficacy, safety and tolerability of high-dose daily CIFN (24 *μ*g) with weight-based ribavirin dosage given with maximal support of patient adherence including blood cell growth factors. Previous clinical trials did not evaluate this high dose of daily CIFN in the treatment of non-responders to pegylated interferon and ribavirin beyond an 8-week induction period [9]. Since the previous data have suggested a possible dose-dependent response to daily CIFN and ribavirin, determining the utility of a higher dosage of daily CIFN with weight-based ribavirin will be important in this group of hepatitis C patients with few efficacious available treatment options.

## 2. Methods

Eligible subjects for our open-label trial were patients infected with chronic hepatitis C genotype 1 previously treated with either pegylated interferon alfa-2a or alfa-2b and ribavirin. In addition, the subjects must have been nonresponsive to prior treatment, defined as the lack of a 2-log drop of hepatitis C viral ribonucleic acid (HCV RNA) from baseline at 12 weeks of therapy or the presence of detectable HCV RNA at 24 weeks of therapy. All subjects must have previously received a weight-based dosage of ribavirin. At least one liver biopsy, consistent with chronic hepatitis C without significant alternative liver disease, must have been performed prior to screening for enrollment into the trial. All subjects, prior to participation, signed a consent form approved by each institutional review board.

This study was an open-label pilot trial. All study subjects were treated with a daily 24 *μ*g dose of CIFN (Infergen-Valeant Pharmaceuticals, Costa Mesa, CA) given subcutaneously and a daily weight-based dose of ribavirin (1000–1200 mg per day). All patients were evaluated at weeks one, two, and four of therapy and at subsequent four week intervals unless additional urgent clinical evaluation was needed. At each visit, subjects underwent physical examination and clinical history detailing adverse events and completion of the Beck Depression Inventory second edition (BDI-II). Treatment was discontinued and the subject considered a treatment failure if the patient failed to achieve early virologic response (EVR) defined as less than a 2-log drop of HCV RNA from baseline at Week 12 of therapy, or had detectable HCV RNA at or after Week 24 of therapy. Sustained viral response (SVR) was defined as an undetectable HCV RNA level using Quest Diagnostics (Madison, NJ) Heptimax and TMA serum assays 24 weeks after completion of 48 weeks of treatment.

Potential subjects for this clinical trial were recruited from three hepatology practices in New York. Compliance with the experimental protocol was assessed by the subjects' self-reported administration of ribavirin and the return of used CIFN vials. In addition, the trial had a data safety monitor (DSM), who assessed the safety of this trial at specified time points during the study. The DSM was an independent physician with extensive clinical experience with treatment of patients with hepatitis C infection. 

The subject's baseline demographics were compared using Student's *t*-test, chi-square and Fisher's exact tests. Response rates between partial and null responders were compared using chi-square and Fisher's exact tests.

## 3. Results

Thirteen subjects (8 men) with a mean age of 55-years were enrolled in this study. The median baseline HCV RNA level was 3,300,000 IU/mL with only two subjects with levels less than 1,000,000 IU/mL. Ten patients had advanced fibrosis (Table 1). During prior initial therapy, ten of the subjects had failed to achieve loss of HCV RNA by 12 weeks of therapy. Three subjects had shown a 2-log decrease in HCV RNA by 12 weeks but had remained HCV RNA detectable at 24 weeks of therapy.

The trial was terminated early due to the occurrence of serious adverse events (*n* = 6) in five patients. These serious adverse events included abdominal wall abscess requiring hospitalization, severe dehydration, and hypotension resulting in metabolic derangements, loss of consciousness, anemia, and ventricular tachycardia. In addition, one subject discontinued therapy due to disabling fatigue. The trial had initially been planned to enroll thirty subjects at this interferon dose. The study termination decision was made by the Principal Investigator on the advice of the DSM after assessing the frequency of serious toxicity while no patient had achieved a sustained loss of HCV RNA. The severity and frequency, of serious adverse events, are evidence that the high-dose aggressive treatment regimen of this study defined an upper dose limit of tolerability for CIFN-ribavirin therapy. 

**Figure 1:**
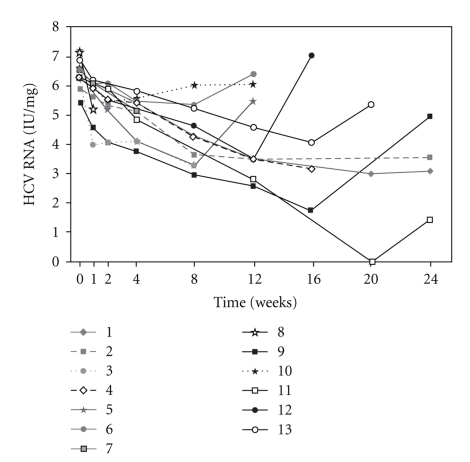
The graph shows the viral kinetics of the 13 subjects during treatment with CIFN and weight-based ribavirin. None of the subjects with an initial drop in HCV RNA, including subject 11 who had transient undetectable HCV RNA at week 20, failed to achieved a sustained loss of HCV RNA.

Subjects were discontinued from treatment, according to protocol, when therapy was judged to be futile or for adverse events. Only four subjects received 24 weeks of treatment. The median maximal decrease of HCV RNA from baseline on treatment was 3,135,000 IU/ml. Nine subjects (69%) had greater than a 2-log drop from baseline HCV RNA while on treatment. One of these had transient loss of HCV but subsequently experienced a viral breakthrough. The four subjects who received 24 weeks of therapy all had partial viral response after 12 weeks of therapy, but had detectable hepatitis C virus at week 24 and therapy was discontinued as they were deemed non-responders (Figure 1).

Three subjects with partial viral response prematurely discontinued therapy due to serious adverse events. There were three subjects still on therapy when the trial was halted. One subject had greater than a 2-log drop in HCV RNA level from baseline by week 8 of therapy, but the HCV RNA level at the time of termination had markedly increased, and there was no longer a 2-log drop in the HCV RNA level from baseline at that point. The second subject did have a greater than 2-log decrease from baseline HCV RNA level after 16 weeks of therapy, but by 20 weeks the hepatitis C viral level had increased over 1-log from the nadir level. The third subject did not have a 2-log decline from baseline viral level after 12 weeks of therapy and was deemed a non-responder. The final three subjects were non-responders to the study medications at the time of discontinuation of therapy due to adverse events.

There were three subjects with greater than a 2-log drop in viral level by four weeks of therapy. One of these subjects could not tolerate therapy and did not complete 12 weeks of therapy. Another was a nonresponder at 24 weeks of therapy. The third subject was still on study medication when the clinical trial was halted, however, while on therapy there was marked increase in HCV RNA level. All three subjects who had greater than a 2-log drop from the baseline viral level after four weeks of therapy were partial responders to previous pegylated interferon and ribavirin therapy compared to none of the prior null responders (*P* < .04).

The most common adverse events were fatigue (*n* = 12) and flu-like symptoms (*n* = 12), anorexia (*n* = 10), anxiety/depression (*n* = 7), and insomnia (*n* = 7). The mean decrease in body weight during the experimental protocol was 8.1 ± 5.8 kilograms. The median increase in BDI-II score was 9 ± 10.1. Serious adverse events (*n* = 6) occurred in five patients and included abdominal wall abscess, severe dehydration, hypotension, and ventricular tachycardia all requiring hospitalization and loss of consciousness and anemia. These events occurred within the first six to 16 weeks of therapy. Two subjects (15%) had their dose of CIFN reduced during the trial from 24 *μ*g to 15 *μ*g daily. Five subjects (38.5%) had a dose reduction of ribavirin and one of the five subjects had ribavirin discontinued. The mean decrease in hemoglobin level from baseline to nadir was 3.6 ± 1.6 g/dL with eight subjects receiving epoetin alpha injections. 

No subject had a decrease in absolute neutrophil count below 500/*μ*L. The mean decrease in WBC level from baseline to nadir was 4,200 ± 1,300/*μ*L. The mean decrease in platelet count from baseline was 104,000 ± 5,200/*μ*L.

## 4. Discussion

Almost half the patients with HCV genotype 1 infection are left with residual infection following treatment with pegylated interferon and ribavirin. Evaluation of currently available agents to optimize therapeutic efficacy is important, particularly for patients with advanced hepatic fibrosis.

Previous studies demonstrate promising SVR rates of 23% to 37% using daily CIFN and ribavirin in the retreatment of non-responders to the combination of standard or pegylated interferon and ribavirin [6–8]. HCV clearance by CIFN appears to be a dose-related response [8, 9, 11]. The efficacy of high-dose CIFN, however, may be limited by tolerability and dose reductions for adverse events. There is no study defining the dose-limiting toxicity of CIFN used with ribavirin. One study, by Böcher et al., compared the efficacy of CIFN and ribavirin with high-dose induction (9 *μ*g daily for 24 weeks followed by 9 *μ*g three times per week for 24 weeks) to lower-dose treatment (CIFN 18 *μ*g three times per week for 12 weeks, followed by 9 *μ*g three times per week for 36 weeks) in those patients who had previously failed treatment with interferon and ribavirin therapy. The week 12 and 24 responses were superior in the high- versus low-dose group (*P* < .05), but the SVR rates were identical at 26%. This suggests that the initial positive effect of the 24 weeks of high-dose daily induction was lost after dose reduction to three times per week treatment [12].

Kaiser etal. from Germany evaluated an induction dose of daily CIFN of 27 *μ*g for four weeks followed by 18 *μ*g for eight weeks versus 18 *μ*g for 12 weeks in 120 patients infected with HCV who were previous non-responders to pegylated interferon and weight-based ribavirin [6]. No ribavirin was given during the induction time period. The SVR rate was 44% in the high-dose group compared to 39% in the lower-dose group. The rate of discontinuation of therapy was 10% in the higher dose arm, and CIFN dose was reduced in 17%. Since this study required significant dose reductions without blood cell growth factor support, the ability to assess the efficacy potential of high-dose daily CIFN was limited.

Another study by Cornberg and colleagues investigated the efficacy of CIFN plus ribavirin in HCV patients who were non-responders to standard interferon and ribavirin [8]. Consensus interferon dosing with 18 *μ*g for the first 8-weeks of treatment resulted in an enhanced first-phase HCV-RNA decay suggesting higher antiviral efficacy of a higher dose of CIFN, but this did not translate to a better SVR, presumably due to dose modifications [8]. Based on the suggestion of a dose-dependent response and a superior response to high-dose CIFN, our trial sought to determine the upper limit of tolerability of CIFN dose in these difficult-to-treat patients. 

Previous partial responders to pegylated interferon and weight-based ribavirin as compared to null responders had better viral kinetic responses with all three partial responders having greater than a 2-log drop in HCV RNA from baseline viral level by week four. This response, however, did not translate into a higher overall efficacy since these subjects could not tolerate continued therapy and viral response did not decline to undetectable levels.

This trial used a sustained high daily dose (24 mg) of CIFN, a treatment regimen which showed promising early results in achieving a decline in HCV RNA levels in a population of subjects resistant to prior therapy. The major factor leading to termination of this study was the occurrence of serious adverse events. Although each event was not unique in patients receiving interferon ribavirin treatment, the occurrence of frequent serious adverse events in a small patient population raised significant concern. The abdominal wall abscess was related to injection site infection. The development of dehydration and hypotension was related to anorexia and severe lassitude. The episode of loss of consciousness may have been related to anorexia and anemia. The observed adverse events, even including cardiac arrhythmias, have been reported previously in patients receiving interferon alfa and ribavirin. The frequency and severity of adverse events, however, effectively determined the upper limit of tolerability of daily consensus interferon to be less than the 24 *μ*g daily dose when used in combination with weight-based ribavirin. This observation is important since the efficacy results of the prior studies left open the possibility of greater efficacy of high-dose CIFN.

Despite use of blood cell growth factors and maximal support of experienced investigators, this high-dose of daily CIFN therapy in combination with a weight-based ribavirin dose was not tolerated due to serious adverse events. Most of the subjects required epoetin alfa injections for therapy-associated anemia. Even in the subjects who could tolerate high-dose CIFN therapy with weight-based ribavirin, sustained loss of HCV was not achieved by 24 weeks. 

We cannot recommend this higher daily 24 *μ*g dose of CIFN in combination with weight-based ribavirin. Our study effectively defines the upper limit of tolerability of daily CIFN dosage when used in combination with ribavirin. Furthermore, this high dosage of CIFN and weight-based ribavirin combination treatment was ineffective in eradicating hepatitis C virus in patients who failed previous pegylated interferon and ribavirin therapy.

## Figures and Tables

**Table 1:** Demographics and baseline data.

Patient Characteristic	Results
Ethnicity:	
Caucasian	61.5%
Hispanic	30.8%
African-American	7.7%

Weight (kilograms)	
Mean ± SEM	84.0 ± 14.9
Histology*	
Stage 1-2	3
Stage 3	5
Stage 4	5

Response to prior treatment (*n*)	
Null response	10
Partial response	3

WBC count (k/*μ*L)	
Mean ± SEM	0.3 ± 1.5
Hemoglobin (g/dL)	
Mean ± SEM	14.6 ± 1.4
Platelet count (k/*μ*L)	
Mean ± SEM	194.9 ± 68.0

*Fibrosis stage defined as modified Ishak score determined as defined by Theise [10].

## Abbreviations

Cifn: consensus interferon
HCV: hepatitis C virus
SVR: sustained viral response
HCV RNA: hepatitis C viral ribonucleic acid
DSM: data safety monitor
WBC: white blood cell
BDI-II: Beck Depression Inventory second edition.
